# Undercover Toxic Ménage à Trois of Amylin, Copper (II) and Metformin in Human Embryonic Kidney Cells

**DOI:** 10.3390/pharmaceutics13060830

**Published:** 2021-06-03

**Authors:** Terenzio Congiu, Mawadda Alghrably, Abdul-Hamid Emwas, Lukasz Jaremko, Joanna I. Lachowicz, Marco Piludu, Monica Piras, Gavino Faa, Giuseppina Pichiri, Mariusz Jaremko, Pierpaolo Coni

**Affiliations:** 1 Department of Medical Sciences and Public Health, University of Cagliari, 09042 Monserrato, Italy; terenzio.congiu@unica.it (T.C.); monipiras@hotmail.com (M.P.); gavinofaa@gmail.com (G.F.); coni@unica.it (P.C.); 2 Division of Biological and Environmental Sciences and Engineering (BESE), King Abdullah University of Science and Technology (KAUST), Thuwal 23955-6900, Saudi Arabia; mawadda.alghrably@kaust.edu.sa (M.A.); lukasz.jaremko@kaust.edu.sa (L.J.); 3 Core Labs, King Abdullah University of Science and Technology (KAUST), Thuwal 23955-6900, Saudi Arabia; abdelhamid.emwas@kaust.edu.sa; 4 Department of Biomedical Sciences, University of Cagliari, 09042 Monserrato, Italy; mpiludu@unica.it; 5 Consorzio Interuniversitario per lo Sviluppo dei Sistemi a Grande Interfase (CSGI), via della Lastruccia 3, 50019 Sesto Fiorentino, Italy

**Keywords:** diabetic nephropathy (DN), metformin, copper, human islet amyloid polypeptide (hIAPP), metal complexes

## Abstract

In recent decades, type 2 diabetes complications have been correlated with amylin aggregation, copper homeostasis and metformin side effects. However, each factor was analyzed separately, and only in some rare cases copper/amylin or copper/metformin complexes were considered. We demonstrate for the first time that binary metformin/amylin and tertiary copper (II)/amylin/metformin complexes of high cellular toxicity are formed and lead to the formation of aggregated multi-level lamellar structures on the cell membrane. Considering the increased concentration of amylin, copper (II) and metformin in kidneys of T2DM patients, our findings on the toxicity of amylin and its adducts may be correlated with diabetic nephropathy development.

## 1. Introduction

Diabetic nephropathy (DN) has one of the highest incidences among the different complications of type 2 diabetes mellitus (T2DM), and in 1997 it accounted for 40% of all new end-stage renal disease (ESRD) cases in the United States, with the cost for treatment of USD 15.6 billion [1]. DN is characterized by progressive albuminuria, with a decline in the glomerular filtration rate that leads to kidney failure, podocyte loss, progressive glomerulosclerosis and, at the final stage, to tubulointerstitial fibrosis [2].

Currently, metformin is the first drug of choice for the treatment of T2DM and is used by at least 120 million people worldwide [3], reaching a market size of USD 280 million in 2019 [4]. Metformin, as a biguanide derivative, is an efficient copper (II) chelator, forming stable Cu(metformin)_2_ complexes (LogK_1_[Cu(metformin)] = 7.17, logK_2_[Cu(metformin)_2_] = 12.30) [5]. In 2012, Logie et al. [6] revealed that the cellular actions of metformin are disrupted by interference with its metal-binding properties. Recently, it was shown that metformin alone [7,8] or in the complex with copper (II) [9,10] can bind to DNA and could exert its anti-tumor activity [11,12].

Metformin has an oral bioavailability of 50–60%, half-life between 2 and 6 h [13] and plasma concentration up to 5 μg/mL (38.8 μmol/L) [14]. Metformin is absorbed incompletely by the intestine (approximately 60%), and the remaining quantities are excreted in faces [15], while about 90% of the absorbed fraction is excreted unchanged in the urine [15]. Up to 2015, metformin was contraindicated by FDA in a large population of T2DM patients mainly due to concerns over metformin-associated lactic acidosis (MALA). In light of new research dismissing the correlation between metformin and MALA [16], and presenting beneficial effects of metformin therapy [17,18,19], the FDA updated its protocols regulating metformin use in T2DM patients with reduced kidney function [20].

Poor glycemic control and diabetes alters the levels of essential trace elements (Cu, Zn, Mg, Mn, Cr, Fe, etc.) by increasing urinary excretion and their concomitant decrease in the blood [21]. In 2005, Cooper et al. [22] showed in clinical studies that Cu homeostasis was altered in diabetic subjects, who demonstrated elevated rates of urinary Cu excretion and a tendency to increased Cu balance compared with control subjects [22].

**Scheme 1:**
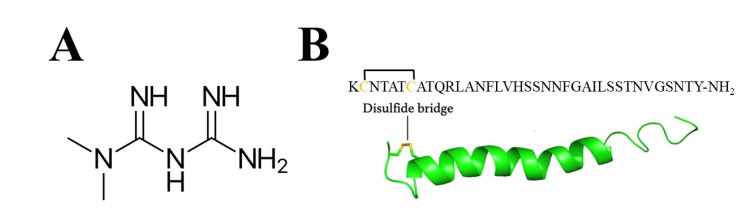
(**A**) Metformin and (**B**) amino acid sequence and NMR structure of hIAPP (PDB: 2KB8). The side chains of Cys [2] and Cys [7] are shown in yellow to illustrate the disulfide bond.

Human islet amyloid polypeptide (hIAPP) (Scheme 1B) is expressed in β-cells and co-secreted with insulin. Extensive reviews of hIAPP function, structure and toxicity have been published in the last decade [23,24]. Human IAPP is one of the most aggregation-prone peptides that interacts with cell membranes, and soluble hIAPP oligomers are more toxic than their aggregated counterparts [25]. Amyloid fibrils are typically 5–15 nm in width, and often many microns long [26,27]. Several studies [28,29,30] verified the high tendency of IAPP to aggregate and misfold into the toxic β-sheet structure. This feature depends on specific amino acids in the primary sequence of the peptide, often associated with the regions located between amino acids 10–20 [31] and/or 20–29 [31,32,33,34,35], with particular attention to His [18,36]. Noteworthily, amylin aggregation is susceptible to changing environmental factors (e.g., pH, which influences the protonation state of the peptide) [37,38] and the presence of other molecules [39,40,41,42] and metal ions [30,43,44]. Zinc (II) and copper (II) are two essential metal ions that can form complexes with amylin [45,46,47] and influence the fibrilization process, although contradictory results can be found in [48,49], most probably due to different experimental conditions. Studies on the potential role of metal ions such as copper on the aggregation of IAPP have intensified over the last two decades, due to its possible biomedical importance [46,50,51]. Interestingly, clinical and epidemiological studies found correlation between T2DM and Alzheimer’s disease, with possible implications of zinc (II) and copper (II) dyshomeostasis [52]. Importantly, rat IAPP differs in six amino acids in the 18–29 region (H^18^→R^18^; F^23^→L^23^; A^25^→P^25^; I^26^→V^26^; S^28^→P^28^; S^29^→P^29^) and does not aggregate [53].

Amylin deposition in the kidneys of patients with diabetic nephropathy was found by Gong et al. [54], and its role was evaluated in different studies [55,56]. Here we investigate, for the first time, the formation of the ternary complex Cu(II)/hIAPP/metformin and its toxicity in human embryonic kidney cells. Chemical and cell culture studies showed that both copper (II) and metformin significantly change the aggregation pattern of amylin, while together they have a synergistic effect that leads to significant morphological changes of the remaining aggregates. Amylin, copper (II) and metformin lead to harmful changes in the cell’s morphology, particularly when the ternary complex is present in the solution.

## 2. Material and Method

Reagents. All reagents (copper (II) nitrate, copper (II) sulphate, metformin, Thioflavin T (ThT), DMSO, 50 mM HEPES buffer) were purchased from Sigma Aldrich. The reagents were of the highest grade commercially available and were used without further purification. Human islet amyloid polypeptide (hIAPP) was purchased from GenScript, with >95% purity (Scheme S1).

EPR measurement. EPR spectroscopy was used in order to study the dynamics and interactions between Cu (II) ions, human amylin and metformin. The EPR spectra were recorded using the procedure previously described [57]. All samples were dissolved in water to the final concentration of 0.3 mM by using stock solution of amylin (10 mg/mL in 100% DMSO), and weighing proper amounts of Cu(NO_3_)_2_ and metformin salts. The pH was fixed at 7.4 by the addition of NaOH and/or HCl. The EPR spectra were recorded using an X-band continuous wave Bruker EMX PLUS spectrometer equipped with a standard resonator for high-sensitivity CW-EPR (Bruker BioSpin, Rheinstetten, Germany).

ThT fluorescence microplate reads. We conducted the ThT fluorescence measurements according to the procedure previously described [57], which is widely accepted in the study of amylin aggregation process [38,58,59]. The working concentrations were as follows: 20 μM thioflavin T (ThT), 2% (*v*/*v*) DMSO (final concentration), and a 50 mM HEPES buffer solution (pH 7.4), different final amylin (from a 10 mg/mL stock in 100% DMSO), metformin and copper concentrations in a final volume of 200 μL. All experiments were conducted at 25 °C. The aggregation kinetics were studied by the use of 96-well flat-bottom black plate (Corning 3915) sealed with transparent film (Duck Brand Crystal Clear Tape, Avon, OH) on on a Synergy 2 Multi-Mode Microplate with excitation set at 485/20 nm and emission at 528/20 nm with a gain equal to 35.

TEM microscopy. Samples containing 100 μM amylin, 100 μM amylin with 100 μM Cu(NO_3_)_2_, 100 μM amylin with 100 μM metformin, and 100 μM amylin with 100 μM copper (II) and 100 μM metformin were incubated at 25 °C and pH 7.4, and aliquots of the reaction were removed after 1 week. The 2 μL solution was applied on the pre-glow discharged EM grids (carbon film on 300 mesh copper grids) and incubated for 1 min. Then, most of the solution was removed with filter paper and stained with 1% uranyl acetate. The air-dried grids were loaded to the microscope (Titan CT, Thermo Fisher Scientific,) for imaging. Most of the images were taken with 0.5 s exposure time at different magnifications.

Mass spectrometry. The spectra and data analyses were performed according to the procedure previously described [57]. We prepared the samples: hIAPP ([hIAPP]_tot_ = 10^−4^ M); 1:1 Cu^2+^:hIAPP stoichiometry ([Cu(II)]_tot_ = [hIAPP]_tot_ = 10^−4^ M); 1:1 metformin:hIAPP stoichiometry ([metformin]_tot_ = [hIAPP]_tot_ = 10^−4^ M); 1:1 Cu(II):metformin:hIAPP stoichiometry ([Cu(II)]_tot_ = [metformin]_tot_ = [hIAPP]_tot_ = 10^−4^ M) in a 50/50 acetonitrile/water mixture at pH 7.4. The analysis was performed using a Micro-TOF Mass Spectrometer from Bruker Daltonics (Germany), equipped with a heated ESI ion source. The mass scan range was set to 100–4000 *m*/*z*, with a resolving power of ~10,000. The *m*/*z* calibration of the TOF analyzer was performed in the positive ESI mode using a solution containing a peptide mixture according to the manufacturer’s guidelines. The ESI was performed with a heated ion source equipped with a metal needle and operated at 4.5 kV. The source vaporizer temperature was adjusted to 300 °C, the exit capillary was set at 400 V.

Cell cultures. Commercial 293T human kidney embryonic cells (catalog code HTL03003) were obtained from the Istituto Nazionale per la Ricerca sul Cancro c/o CBA (ICLC, Genova). The cell cultures were prepared following previously described procedures [60]. After 24 h of growth with the complete medium, the cells were grown in the presence of different experimental samples: hIAPP (daily prepared solution; different concentration conditions), metformin 4.7 μM, copper (II) sulphate 4.7 μM, hIAPP with copper (II) sulphate (4.7 μM; 1:1 molar ratio) and hIAPP, copper and metformin (4.7 μM; 1:1:1 molar ratio).

Fluorescence microscopy. Zoes microscopy was used for live cell imaging at bright field and green channel (λ_Ex_ 480 ± 17; λ_Em_ 517 ± 23). Thioflavin T (ThT) staining was prepared as follows: 1 mM solution of ThT in buffer (50 mM phosphate; 100 mM NaCl; pH 7.4) was diluted (1:100) with the “measurement buffer” (50 mM glycine at pH 8.5) on the day of measurement; 5 μL of diluted ThT was added to the cell growth medium (final ThT concentration 0.025 μM). The images were collected after 2 h incubation with ThT marker. The images were processed with ImageJ^®^ software (https://imagej.nih.gov/ij/index.html, accessed on 21 January 2021), and the intensity of the fluorescence was measured with ImageJ tools.

Optic microscopy. Cell samples growing in different experimental conditions were stained with the classical hematoxylin/eosin staining technique [61], whereas digital image acquisition was performed on an Olympus BH2 series system light microscope.

SEM microscopy. Samples were collected and fixed with 4% buffered formaldehyde and then washed three times in PBS. For SEM analysis, specimens were post-fixed for 1 h in a solution of 1% osmium tetroxide and 1.25% potassium ferrous-cyanide and then washed three times in PBS. In the next step, the samples were dehydrated (ethanol series, critical point drying (CPD) in CO_2_). The samples, mounted on carbonated stubs and gold-coated in a sputter coater, were observed with a SEM microscope (ZEISS, Sigma 500 VP).

Quantitative cell analysis. The cell concentration, viability and size were evaluated using the LunaFL Cell Counter (www.logosbio.com, accessed on 21 January 2021) as indicated in the manufacturer’s user manual. Briefly, cell samples mixed 1:1 with 0.4% Trypan Blue solution, supplied in the kit reagents, were counted in the Luna cell counting chamber slide within 1–3 min. Results were analyzed as cell concentration, percent of cell viability and cell size. To obtain accurate data, each experiment was repeated two times, and at least three measurements were acquired for each sample. The standard deviation bars (Figure S8) represent the errors of all measurements for each experiment. Cells growing in the medium containing copper ions (final concentration 4.74 μM) showed cell concentration and viability similar to that of the control sample (normal growing conditions), while the cell size was slightly reduced. The addition of metformin (final concentration 4.74 μM) to the cell growing medium reduced the cell concentration and cell viability, while the cell size remained similar to that of control sample. The presence of amylin (final concentration 4.74 μM) in the cell growing medium leads to the lower cell concentration and viability, while the cell size remains similar to that of the control sample. The presence of amylin equimolar complexes with coper and/or metformin (final concentration 4.74 μM) exert its toxicity at a lower cell concentration, viability and cell size. The lowest values of all three parameters were found for the cells growing with amylin, copper and metformin.

## 3. Results 

### 3.1. In Vitro Copper, Metformin and Amylin Interactions

**Figure 1:**
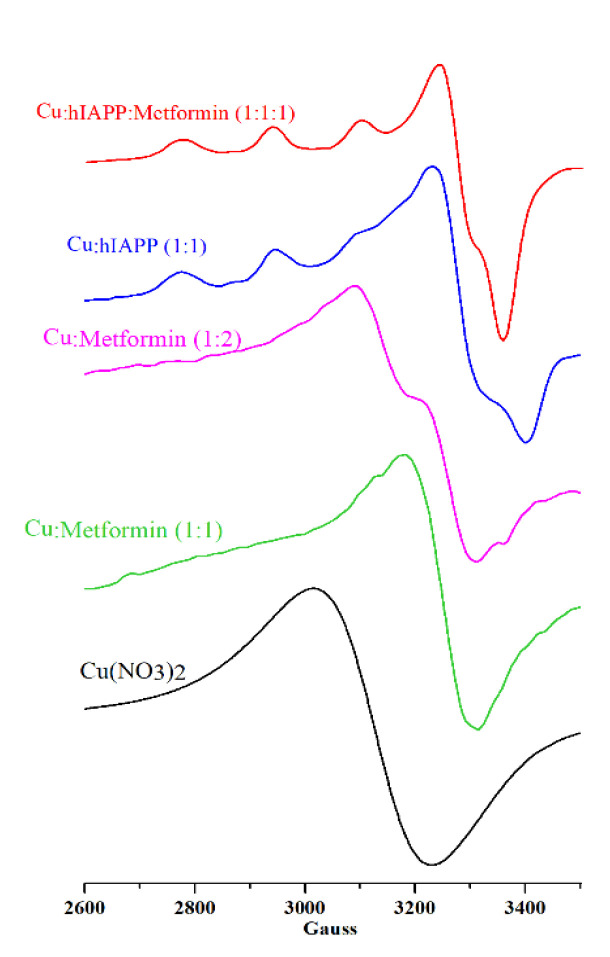
EPR spectra of Cu(NO_3_)_2_ (0.3 mM; black line), Cu(II)/metformin (0.3 mM; molar ratio 1:1 green line; molar ratio 1:2 magenta line), Cu(II)/hIAPP (0.3 mM; molar ratio 1:1; blue line) and Cu(II)/hIAPP/metformin (0.3 mM; molar ratio 1:1; red line) at pH 7.4 in water.

Electron paramagnetic resonance (EPR) probes the structure of copper binding sites [62] and permits the establishment of ligands in close proximity to the metal center and the structure of the formed complexes. The copper (II) complexes with metformin were previously studied by means of EPR spectroscopy [5,63] showing the square-planar geometry of the Cu(metformin)_2_ complex. In Figure 1, two solutions with 1:1 and 1:2 metal:metformin molar ratios are presented. The solution containing 1:2 metal:metformin molar ration has the shape of EPR signals similar to that reported for the Cu(metformin)_2_ complex [5], while the equimolar solution has slightly higher g value and different shape. Such differences could be assigned to the formation of different stoichiometry and geometry complexes, but further analysis is needed.

Recently, EPR spectra of hIAPP complexes with copper (II) in different experimental conditions (pH and metal:peptide molar ratio) were extensively studied by Seal et al. [64]. They showed that copper (II) forms with amylin in 1:1 stoichiometry complexes, and different metal binding sites are placed within the amylin [1,2,3,4,5,6,7,8,9,10,11,12,13,14,15,16,17,18,19] fragment. The metal binding mode depends on the pH, and in the physiological range two types of complexes (named I and II) can be distinguished by EPR spectra. Complex I (g_‖_ = 2.17, g_┴_ = 2.03, A_‖_ = 195; 4N coordination mode from the Peisach–Blumberg plot [62]) is predominant at pH 8.0, and based on the combined EPR, UV–vis and CD data the authors concluded that the metal ion is coordinated by N-terminal amine (N-donor), amidate (N-donor), but could be also coordinated by hydroxide (HO^–^) and carbonyl (O-donor) groups. Complex II is formed mainly at pH 6.0 (g_‖_ = 2.20, g_┴_ = 2.04, A_‖_ = 162; 3N1O coordination mode from the Peisach–Blumberg plot [62]), and the copper ion is coordinated by histidine, amidate, amine and O-donors from amide carbonyl or H_2_O.

More structural data of Cu(II)/amylin complex can be found in the paper of Magrì et al. [47], who studied copper (II) coordination by hIAPP [14,15,16,17,18,19,20,21,22] and hIAPP [17,18,19,20,21,22,23,24,25,26,27,28,29] PEG-ylated fragments by means of EPR, UV–vis and CD. They showed that at physiological pH (7–8) the metal ion is coordinated by nitrogen atom of His [18] and three deprotonated amides of the preceding peptide bonds.

In our experimental conditions, the EPR spectra (Figure 1; Table 1) of Cu(II)/hIAPP (blue), Cu(II)/metformin 1:1 molar ratio (green), Cu(II)/metformin 1:2 molar ratio (magenta) and Cu(II)/hIAPP/metformin (red) were distinctly different from free Cu(II) (black) in solution at the same pH, and each spectrum represents a different signal pattern. 

The EPR signal of the Cu(II)/hIAPP complex (Figure 1, blue) with g_‖_ = 2.23 and A_‖_ = 171 G corresponds to the signal previously reported by Seal et al. [64] and assigned to the tetragonal Cu/amylin complexes having a dx^2^ − y^2^ ground state in a square-planar or square-pyramidal geometry with a weak axial ligand. Moreover, the comparison of data presented here with that of Seal et al. (Table 1) and Magrì et al. (PEG-hIAPP [14,15,16,17,18,19,20,21,22]; pH 8; g_‖_ 2.217, A_‖_178; PEG-hIAPP [17,18,19,20,21,22,23,24,25,26,27,28,29]; pH 8; g_‖_ 2.225, A_‖_161) confirm the main presence of the complex with the coordination mode 4N at pH 7.4. The addition of metformin to the solution containing copper ions and hIAPP leads to the formation of a complex, with a lower A_‖_ value with respect to the binary Cu(II)/hIAPP complex, and better defined signal at ~3100 G. This new complex could be assigned to the ternary Cu(II)/hIAPP/metformin adduct, and the coordination mode from the Peisach−Blumberg plot [62] can be associated to 3N1O. Following Peisach–Blumberg analysis [62], the change of only the A_‖_ value (with respect to the Cu(II)/hIAPP complex) suggests that the overall charge of both complexes remains the same. It is likely that metformin contributes to the metal-coordination center with one or two nitrogen atoms, while the remaining metal binding sites are associated to hIAPP, but in-depth analysis of the metal binding sites and the geometry of the complex is out of the scope of this paper. 

The complex formation between amylin, metformin and copper (II) ions was studied by means of ESI-MS spectrometry (Supplementary Material Figure S5). In the solution containing hIAPP and copper ions, there are signals at 1322.6102 and 1983.4039 *m*/*z* (Figure S4B), which can be assigned to the [M+Cu(II)+H]^3+^ and [M+Cu(II)]^2+^ complexes (M = hIAPP). The amylin/metformin adduct is represented by the signal at 1342.9244 *m*/*z* (Figure S4C) in the solution of amylin and metformin. The exact nature of amylin/metformin adduct is unknown, but it is likely that the biguanide chain of metformin forms hydrogen bonds (–NH^…^) with the amylin backbone and/or amino acid side chains. As shown further by the TEM experiments, the formation of amylin/metformin adducts significantly changes the morphology of amylin aggregates. The EPR data showed that in the ternary Cu(II)/hIAPP/metformin complex metformin binds directly to the metal center, but it is also possible that metformin/amylin interactions occur along the peptide backbone and/or amino acid side chains. Nevertheless, in the mass spectra of the solution containing hIAPP, metformin and metal ions (Figure S4D) there is no signal associated to the tertiary copper/amylin/metformin adduct, probably due low stability at 300 °C experimental conditions, and only the signal at 1322.6102 *m*/*z* of [M+Cu(II)+H]^3+^ can be observed.

The fluorescent dye thioflavin T (ThT) is commonly used to measure the kinetics of protein assembly in vitro. It associates rapidly (within seconds) with aggregated fibrils, giving rise to enhanced fluorescence emission [65]. ThT is supposed to interact specifically with the crossed-β-sheet structures, and the fluorescence enhancement of ThT depends on the structure of the aggregated state of the amyloid peptides [65]. Moreover, polycationic materials fail to interact or impede interaction with the amyloid peptides [65]. Aware of advantages and limitations of ThT, we investigated the influence of copper (II) ions, metformin and both copper (II) and metformin on amylin aggregation.

**Figure 2:**
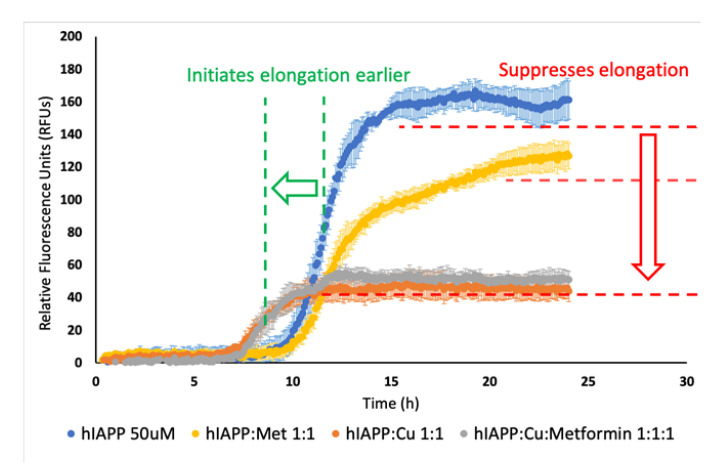
Effect of time and Cu^2+^ (50 μM), metformin (50 μM) and Cu^2+^/metformin (50 μM) on hIAPP (50 μM) aggregation in a 50 mM HEPES-buffered solution pH 7.4, at 25 °C. ThT (20 μM) was used as a fluorescence marker of the aggregation process.

In a series of experiments (Figure S1), we observed that the fluorescence intensity was directly proportional to the concentration of the reagents. Solutions containing higher concentrations of the reagents showed higher fluorescence intensity, which could be associated to lower amylin solubility and increased formation of aggregates. Noteworthily, Cu(II)/hIAPP and Cu(II)/hIAPP/Metformin solutions had the same fluorescence intensity, which was much lower than in the solution containing only hIAPP. The elongation phase of Cu(II)/hIAPP and Cu(II)/hIAPP/metformin solutions started faster and was shorter in time compared to the same phase of the hIAPP solution. In the experiment using 50 μM hIAPP, copper ions and metformin (Figure 2), we observed that the presence of metformin in the solution of hIAPP lowered the fluorescence intensity, but it did not significantly influence the elongation phase.

**Figure 3:**
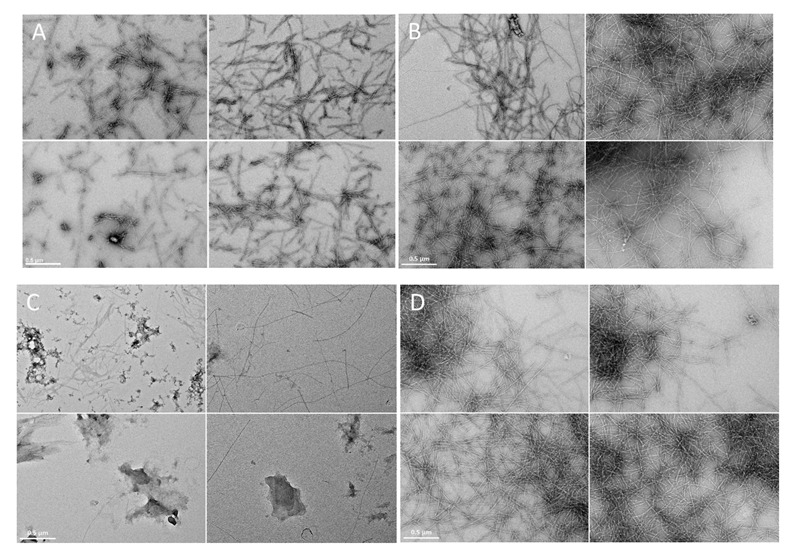
Electron micrographs (four representative images) of amylin fibrils at pH 7.4 in the water solution containing: (**A**) amylin (100 μM); (**B**) Cu(II)/amylin (100 μM; molar ratio 1:1); (**C**) metformin/amylin (100 μM; molar ratio 1:1); (**D**) Cu(II)/amylin/metformin (100 μM; molar ratio 1:1:1). Incubation time 7 days, T = 25 °C. The scale bar (0.5 μm) is equal for all images.

In order to better assess the ThT fluorescence results, microscopic observations of protein aggregates were prepared with TEM microscopy. It was observed at micrometer resolution that amylin formed fibril structure aggregates (Figure 3A), which were found previously with the same experimental conditions by Jha et al. [38]. The addition of copper ions to the amylin solution led to formation of Cu(II)/hIAPP complexes [64], and the formation of longer fibril structures seemingly increased (Figure 3B). Metformin and hIAPP form a complex, which to our knowledge has not been previously described. The presence of metformin in the hIAPP solution significantly reduced the formation of fibril structures (Figure 3C) and led to the formation of amorphous aggregates. Incubation of copper ions together with hIAPP and metformin led to the formation of the ternary Cu(II)/hIAPP/metformin adduct and significantly increased the formation of fibril structures of high electron density (Figure 3D).

ThT fluorescence experiments (Figure S2), conducted on the samples prepared under the same experimental conditions as the TEM samples, showed a lower fluorescence intensity for the Cu(II)/hIAPP and Cu(II)/hIAPP/metformin complexes, compared to the hIAPP sample. Noteworthily, the highest fluorescence intensity was observed in the sample of the metformin/hIAPP adduct, and such a high fluorescence intensity after 7 days of incubation needs further investigation. As shown by TEM observations of the formed aggregates, the decrease/increase in ThT fluorescence intensity can be associated with the change in aggregate morphology, rather than decrease/increase in the aggregation process rate.

### 3.2. Toxicity of Amylin Adducts with Copper and/or Metformin in 239T Cells

**Figure 4:**
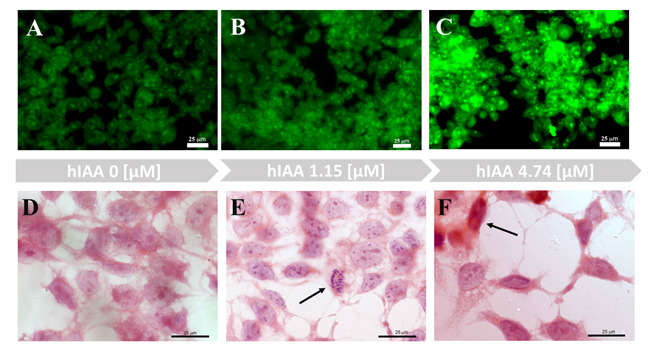
Imaging of 293T cells growing in different hIAPP concentrations. (**A**–**C**) Live cell images of ThT (0.025 μM) staining (2 h). (**D**–**F**) Hematoxylin and eosin staining.

Human embryonic 293T kidney cells were used recently for in vitro studies on the mechanisms of nephropathy pathology [66,67,68]. In our experimental studies, 293T cells were used to investigate cell morphology after exposure to hIAPP and its complexes with copper ions and/or metformin. In the series of experiments (Figure 4 and Figure S5), we observed that growing concentrations of hIAPP in the cell medium led to reduced cell numbers and changes in the morphology of the remaining cells, compared to the control sample. The hIAPP concentration that did not interfere with cell number, but led to morphological changes in half of the growing cells, was 4.74 μM. The ThT staining (Figure 4A–C) of the 293T cells in medium with different hIAPP concentrations showed that cells treated with 4.74 μM hIAPP (Figure 4C) had higher fluorescence intensity with respect to the control sample and cells treated with 1.15 μM hIAPP. 

Hematoxylin and eosin staining permits observation of the morphology of 293T cells growing in medium with different hIAPP concentrations (Figure 4D–F). A low hIAPP concentration (1.15 μM) did not interfere with normal cell activity or mitotic processes (Figure 4E, arrow). In contrast, a fourfold increase in hIAPP concentration led to morphological changes of the cells (Figure 4F) and significantly reduced the number of cellular expansions. Some cells also displayed apoptotic phenotypes (Figure 4F, arrow).

ThT fluorescence staining was used for live-cell imaging of the 293T cells growing in the absence or presence of amylin and its adducts with metal ions and metformin (Figure S6). ThT reacted immediately with live cells and showed low fluorescence intensity in the control sample. In the presence of amylin (Figure S6B), the fluorescence intensity slightly increased. Moreover, cell numbers in the culture with hIAPP were reduced. In the cell culture containing amylin and copper (II) ions (Figure S6C), the fluorescence intensity was slightly lower than in the culture only with amylin, while the cell number remained similar. Cell numbers were drastically reduced in samples containing metformin (Figure S6D,E) and had lower fluorescence intensity compared to the sample with amylin only. 

The general trend of fluorescence intensity in the live-cell cultures with amylin and its adducts with copper (II) and/or metformin was similar to that observed in vitro without cells (Figures S1 and S2). Nevertheless, we should treat the data with caution due to the high fluorescence intensity oscillation among the same samples. Thioflavin T staining lacks specificity versus amylin, and its fluorescence increases also upon binding to β-sheet-rich peptides [69]. It also cannot be excluded that stress conditions in experimental samples led to the formation of different amyloid structures that interact with ThT and increase the fluorescence intensity.

Metabolic and molecular disorders, which are induced by risk factors of T2D, might promote the accumulation of soluble IAPP-related molecules and toxic IAPP oligomers in β-cells, impairing function and reducing the mass though disruption of cell membranes [25]. Human IAPP interacts with negatively charged membranes, and this interaction can drastically accelerate misfolding, which is a prerequisite for hIAPP toxicity [70]. It has been speculated that the α-helical structure might be important for hIAPP membrane interactions, leading to high local concentrations and, in turn, promoting intermolecular β-sheet formation and IAPP oligomers, in which toxic oligomers disrupt cell membranes in β-cells. A recent study showed that fresh and oligomeric hIAPP, as well as mature amyloid, enhanced membrane fluidity and reduced cell viability [71]. 

**Figure 5:**
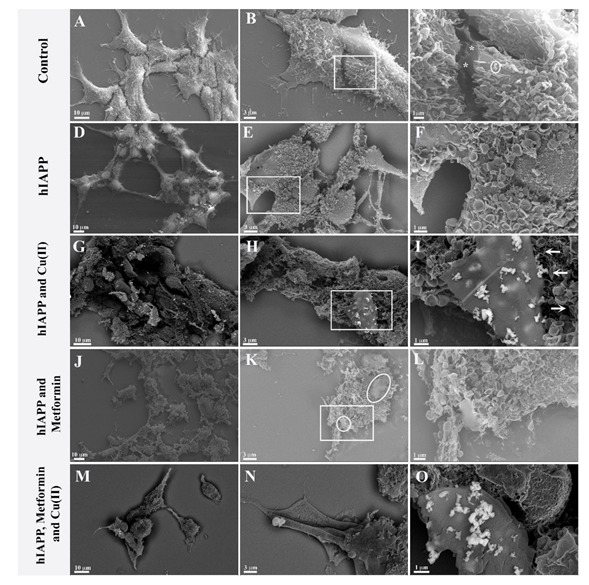
Ultrastructural images of 293 T cells growing 24 h in (**A**–**C**) normal growing conditions, (*) intracellular contacts with the junction points; (**D**–**F**) with 4.74 μM hIAPP; (**G**–**I**) with 4.74 μM hIAPP and 4.74 μM copper ions; (**J**–**L**) with 4.74 μM hIAPP and 4.74 μM metformin; (**M**–**O**) with 4.74 μM hIAPP, 4.74 μM copper and 4.74 μM metformin.

In order to investigate possible interactions with the cellular membrane of hIAPP and its adducts with metformin and/or copper (II) ions, quantitative cell analysis (Supplementary Material Figure S8) and qualitative SEM analyses were performed. Figure 5A–C presents SEM imagining, where we observed the control 293T cell culture, which was confluent with a homogeneous cell distribution. The cells adhered well and formed numerous cytoplasmic expansions. Multiple microvilli structures were observed on the cell surface (Figure 5C), some occasionally with an ‘ear-like’ shape (Figure 5C circle). In addition, intracellular contacts could be observed with the junction points (Figure 5C asterisk).

In contrast, cells cultured with amylin (Figure 5D–F) did not grow to confluence and had a ‘suffering’ appearance: the cells were unevenly distributed. Moreover, the cells retained only residual intra-cellular junctions (Figure 5D), and elongated protrusions were observed (Figure 5D). Furthermore, densification of the cytoplasm was observed, and the nuclei were well evident. The microvilli had mostly an ear-like shape distributed evenly on the surface of the cells. The amylin aggregates, which were previously observed in vitro (Figure 3), were not observed in the cell culture. In recent studies, Tomasello et al. [72] showed that hIAPP [1,2,3,4,5,6,7,8,9,10,11,12,13,14,15,16,17,18,19,20,21,22,23,24,25,26,27,28,29,30,31,32,33,34,35,36,37] exerted toxic effects on rat insulinoma cells after 62 h incubation with 60 μM peptide, modifying cell morphology and leading to the formation of elongated protrusions. Fluorescent labelling of hIAPP [1,2,3,4,5,6,7,8,9,10,11,12,13,14,15,16,17,18,19,20,21,22,23,24,25,26,27,28,29,30,31,32,33,34,35,36,37] showed peptide localization not only on the cell membrane but also in mitochondria and the cytoplasm. Importantly, the same results were observed with the hIAPP [17,18,19,20,21,22,23,24,25,26,27,28,29] fragment, which in vitro does not form β-sheet structures and aggregates. In our studies, the 293T human kidney embryo cells showed higher sensitivity to amylin toxicity with respect to rat insulinoma cells, and the toxic effect could be observed already after 24 h with 4.74 μM peptide. Similarly to Tomasello et al., we observed significant morphological changes of the cells growing in the presence of amylin, and the formation of elongated protrusions, but we have not observed amylin aggregates on the cell surface. As previously reported by Tomasello et al., amylin could be localized inside the cell, where it exerts its toxic effect.

Cells cultured with amylin and copper ions (Figure 5G–I) showed greater distress than cell cultures with amylin alone. Cellular expansions were not observed, and the nuclei were no longer prominent. The cell membrane was altered, while the ‘ear-like’ microvilli were unevenly distributed. Multilayer lamellar compact aggregates were observed (Figure 5H,I) and could be associated with amylin aggregates. Furthermore, the backscatter images showed the presence of copper ions only in the vicinity of amylin aggregates (Figure 5H,I). It is important to note that the multi-layer formations of the aggregate are at different stages of development. Some fibrillar structures could be observed (Figure 5I arrow) albeit at lower frequencies and without copper ions.

Cells grown in the presence of both amylin and metformin (Figure 5J–L) were more severely affected than cells grown with amylin alone. The nuclei were not evident, and it was not possible to recognize cellular organization. Amorphous aggregates (Figure 5K circle) with a morphological structure different from amylin aggregates formed in the presence of copper (Figure 5G–I) could be observed. 

**Figure 6:**
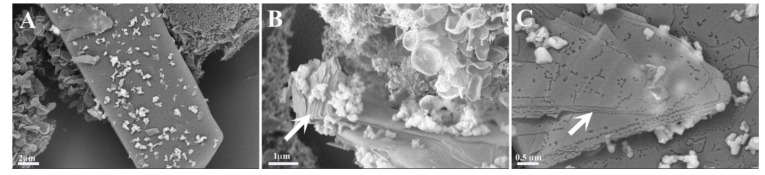
Ultrastructural images with enlargement of multilamellar aggregates (arrows) formed on the cell surface of 293T cells grown 24 h in the presence of amylin (4.74 μM), copper (4.74 μM) and metformin (4.74 μM). (**A**–**C**) present different sections of the analyzed sample.

**Scheme 2:**
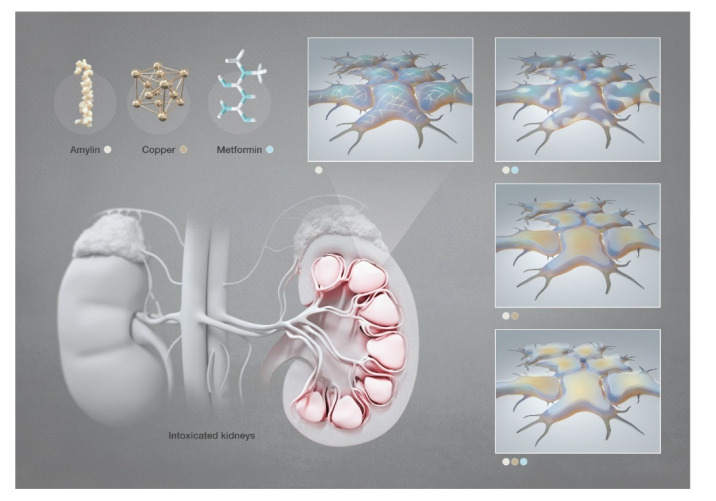
Graphical representation of the SEM investigation of hIAPP aggregates in human embryonic kidney cells in the presence of copper and/or metformin. [Images produced by KAUST scientific illustrators. This image is original and designed specifically for the targeted publication. It should not be cropped, distorted or in any way edited without the expressed consent of Research Publication Services. Because KAUST owns the copyright of the original image, you may cause a copyright conflict between the journal and KAUST if you modify the illustration in any way before submitting it to the journal. Any other use of the image apart from your paper (e.g., in a presentation, poster, or website) should be accompanied by credits to the journal in which it is published and to the illustrator as follows: Heno Hwang/KAUST].

Cells cultured with amylin, copper and metformin were evidently in distress (Figure 5M–O). We observed cells overlapping, and, in some cells, the nuclei could be observed. Long cell expansions were observed, but with few junctions. The copper ions were clearly visible and strictly correlated with the presence of composed lamellar structures. The lamellar structures were over dozens of micrometers long (Figure 6A) and formed multilayer lamellar structures, which appeared on the cell surface. Figure 6B shows the formation of multilamellar structures (arrow) at different stages. The position of the aggregate, which perfectly fits to the cell membrane, is testimony to the contemporary growth of the amyloid with the cell. Figure 6C reveals that each layer (arrow) of the aggregate is accompanied by the copper ions. The graphical summary of SEM investigations is presented in Scheme 2.

The recent findings of amylin aggregation in the brain and possible correlation with neurodegenerative pathologies [73] and metal ion homeostasis led to a thorough analysis of the amylin aggregates at the molecular level [74]. Although metformin interactions with metal ions are well ascertained, up to now there have been no studies on the possible interactions of metformin with amylin and copper ions.

## 4. Conclusions

Our studies show clearly that metformin forms adducts with amylin and, in vitro, leads to the formation of aggregates with a morphology distinct from that of amylin fibrils. The metformin/amylin adducts induce high toxicity in the cell culture, where amorphous aggregates can be observed on the cell membrane.

Copper (II) ions form stable complexes with amylin and significantly change the morphology of amylin aggregates in vitro. The well-known fibrillar aggregates of amylin are transformed into composed lamellar structures in the presence of copper ions in cell culture media. The copper/amylin aggregates grow on the cell membrane leading to its disruption.

Toxic effects of copper (II)/amylin adducts on cells is enhanced by the presence of metformin and the successive formation of a ternary copper (II)/amylin/metformin complex. The contemporary presence of these molecules leads to the low cell number in the culture, changes in cell morphology and the formation of numerous lamellar aggregates on the cell membrane.

The cellular toxicity of copper (II)/amylin and copper (II)/amylin/metformin complexes should be examined in vivo in order to exclude or confirm possible metformin side effects in diabetic patients.

## Figures and Tables

**Table 1:** EPR parameters of the copper complexes with hIAPP and/or metformin compared with published data.

Assumed Composition of the Complex	Obtained EPR Results			Assumed Composition of the Complex	Literature EPR Results		
	A_‖_ ± 3, G	g_‖_ ± 0.05	g_┴_ ± 0.005		A_‖_	g_‖_	g_┴_
Cu:hIAPP	171	2.23	2.03	Cu:hIAPP pH 8.0 [64]	195	2.17	2.03
				Cu:hIAPP pH 6.0 [64]	162	2.20	2.04
Cu:Metformin (1:2)	-	-	2.06	Cu:Metformin [63]	-	2.179	2.031
Cu:Metformin (1:1)	-	-	2.08				
Cu:hIAPP:Metformin	165	2.23	2.04	Cu:hIAPP:Metformin	-	-	-

## Data Availability

All data generated or analyzed during this study are included in the published article (and its online Supplementary Materials).

## Supplementary Materials

The following are available online at https://www.mdpi.com/article/10.3390/pharmaceutics13060830/s1, Scheme S1. Chromatogram of hIAPP sample delivered by GenScript. Figure S1. Effect of Cu (II) and Cu (II)/Metformin on hIAPP (in a 50 mM HEPES-buffered solution pH 7.4) fluorescence with ThT (20 μM) at 25 °C in different concentration conditions: (A) 50 μM; (B) 40 μM; (C) 30 μM and (D) 20 μM. Figure S2. Effect of Cu (II) (100 μM) and Cu (II)/Metformin (100 μM; 1:1 molar ratio) on hIAPP (100 μM; in a 50 mM HEPES-buffered solution pH 7.4) fluorescence with ThT (20 μM) at 25 °C. Figure S3. Effect of Cu (II) (100 μM) and Metformin (100 μM) on ThT (20 μM) fluorescence at 25 °C. Figure S4. Full Scan Mass spectra acquired using the ESI-(+)-Micro-TOF-MS instrument. (A) hlAPPI solution. Signals at 976.7343; 1301.9733 and 1952.4508 *m*/*z* are assigned to hIAPP (M) at different protonation states: [M+4H]^4+^; [M+3H]^3+^; [M+2H]^2+^; respectively. (B) hlAPPI/Cu(II) solution. Signals at 1322.6102 and 1983.4039 *m*/*z* are assigned to copper complexes: [M+Cu(II)+H]^3+^ and [M+Cu(II)]^2+^, respectively. (C) Metformin/hIAPP solution. Signal at 1342.9244 *m*/*z* is assigned to metformin (Met) adduct: [M+Met-3H]^3+^. (D) Cu(II)/hIAPP/Metformin solution. Signal 1322.6102 is assigned to copper complexes: [M+Cu(II)+H]^3+^. Insets: comparison of experimental (up) and simulated (down) isotopic distributions. Figure S5. Live cell images of 293T cell cultures incubated 24 h with growing concentration of hIAPP. Figure S6. ThT live-cell fluorescence staining of 293T cells growing (A) in normal conditions; (B) with hIAPP (4.74 μM); (C) with hIAPP (4.74 μM) and copper ions (4.74 μM); (D) with hIAPP (4.74 μM) and metformin (4.74 μM); (E) with hIAPP (4.74 μM), copper ions (4.74 μM) and metformin (4.74 μM) for 24 h. The mean, min and max fluorescence intensity of each experimental sample (down) was calculated with ImageJ software. Figure S7. The graphical representation of LunaFL Cell Counter results of cell concentration (A), cell viability (B) and cell size (C).
