# Supplementary material for: Undercover Toxic Ménage à Trois of Amylin, Copper (II) and Metformin in Human Embryonic Kidney Cells
Source: Pharmaceutics. 2021 Jun 3;13(6):830. doi: 10.3390/pharmaceutics13060830 (PMC8229594; doi:10.3390/pharmaceutics13060830)
Supplement: Supplementary file 1 [file pharmaceutics-13-00830-s001.zip › pharmaceutics-1223914-supplementary.pdf]

pharmaceutics

MDPI

Supplementary Materials: Undercover Toxic Ménage à Trois of Amylin, Copper (II) and Metformin in Human Embryonic Kidney Cells

Terenzio Congiu, Mawadda N. Alghrably, Abdul-Hamid Emwas, Lukasz Jaremko, Joanna I. Lachowicz, Marco Piludu, Monica Piras, Gavino Faa, Giuseppina Pichiri, Mariusz Jaremko and Pierpaolo Coni

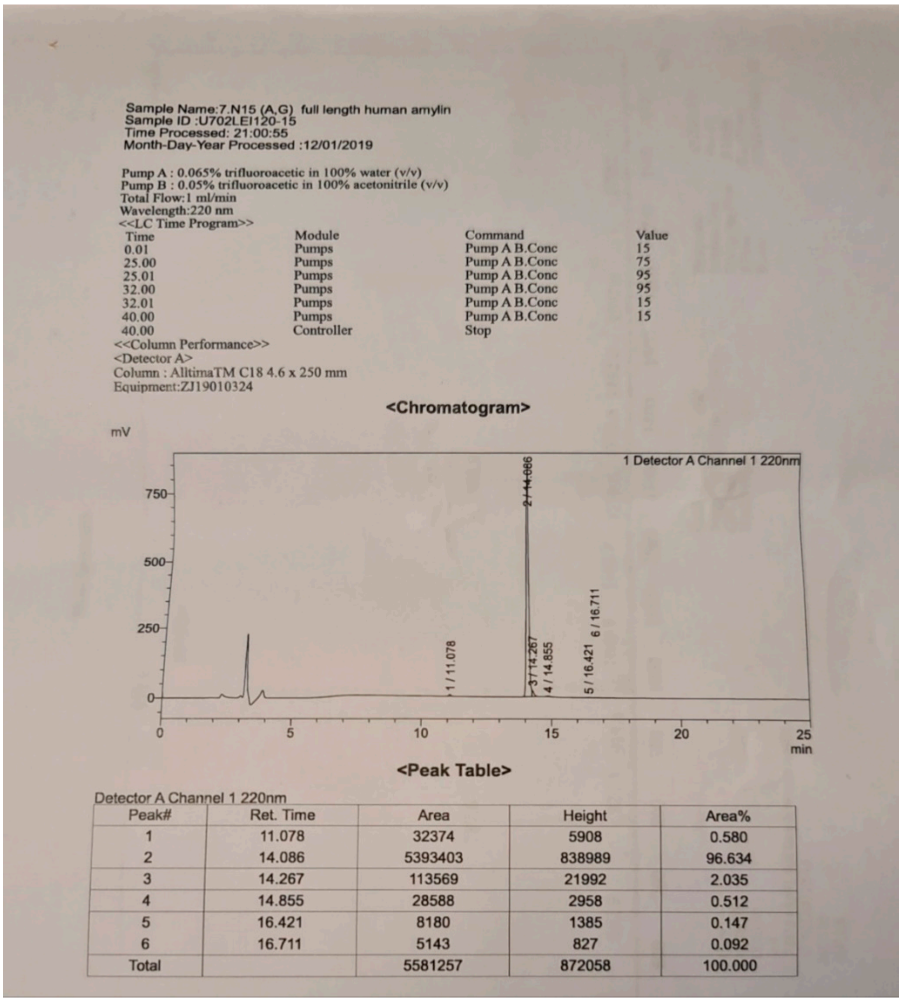

Sample Name:7.N15 (A,G) full length human amylin
Sample ID :U702LEI120-15
Time Processed: 21:00:55
Month-Day-Year Processed :12/01/2019

Pump A : 0.065% trifluoroacetic in 100% water (v/v)
Pump B : 0.05% trifluoroacetic in 100% acetonitrile (v/v)
Total Flow:1 ml/min
Wavelength:220 nm
<<LC Time Program>>

Time	Module	Command	Value
0.01	Pumps	Pump A B.Conc	15
25.00	Pumps	Pump A B.Conc	75
25.01	Pumps	Pump A B.Conc	95
32.00	Pumps	Pump A B.Conc	95
32.01	Pumps	Pump A B.Conc	15
40.00	Pumps	Pump A B.Conc	15
40.00	Controller	Stop	

<<Column Performance>>
<Detector A>
Column : AlltimaTM C18 4.6 x 250 mm
Equipment:ZJ19010324

<Chromatogram>

<Peak Table>

Detector A Channel 1 220nm

Peak#	Ret. Time	Area	Height	Area%
1	11.078	32374	5908	0.580
2	14.086	5393403	838989	96.634
3	14.267	113569	21992	2.035
4	14.855	28588	2958	0.512
5	16.421	8180	1385	0.147
6	16.711	5143	827	0.092
Total		5581257	872058	100.000

Scheme S1. Chromatogram of hIAPP sample delivered by GenScript.

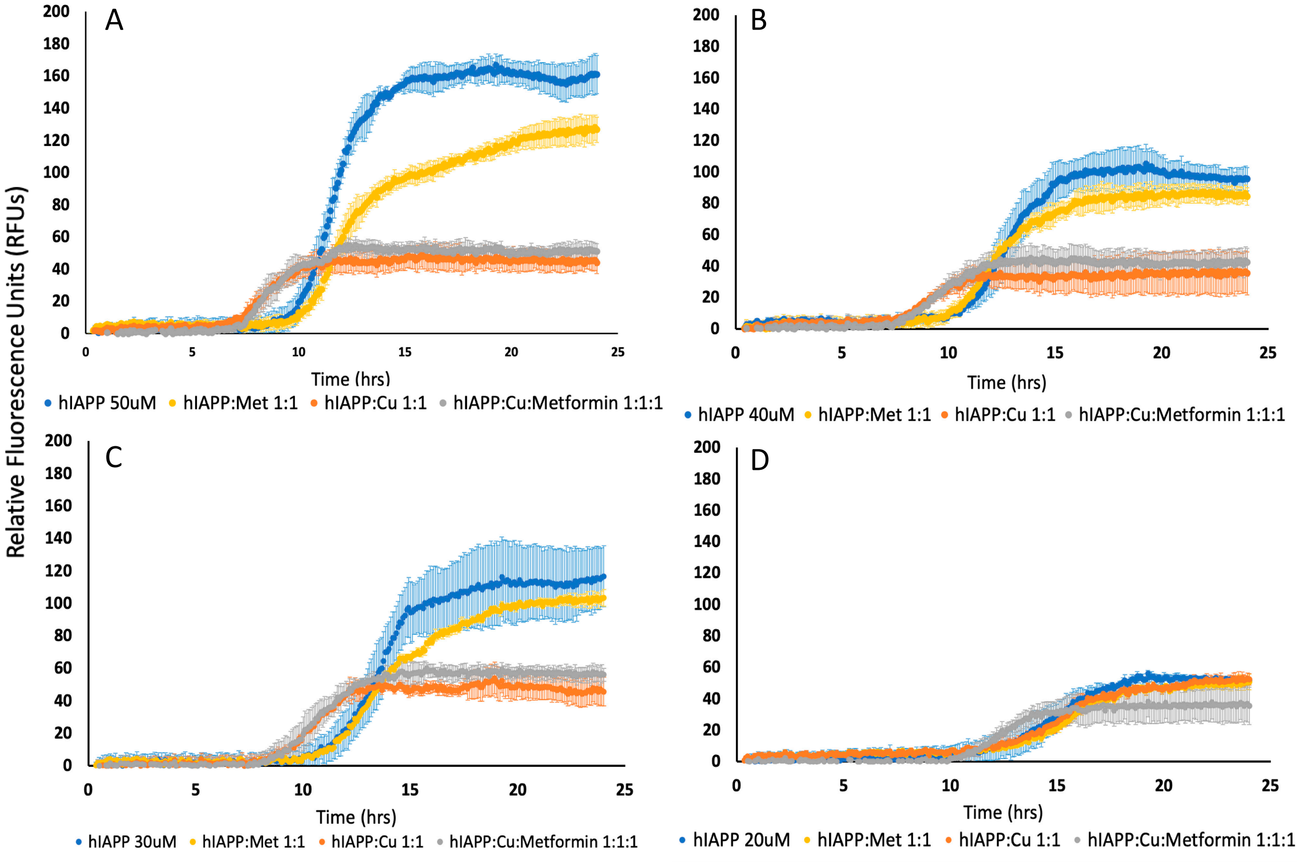

Figure S1. Effect of Cu (II) and Cu (II)/Metformin on hIAPP (in a 50 mM HEPES-buffered solution pH 7.4) fluorescence with ThT (20 μM) at 25 °C in different concentration conditions: (**A**) 50 μM; (**B**) 40 μM; (**C**) 30 μM and (**D**) 20 μM.

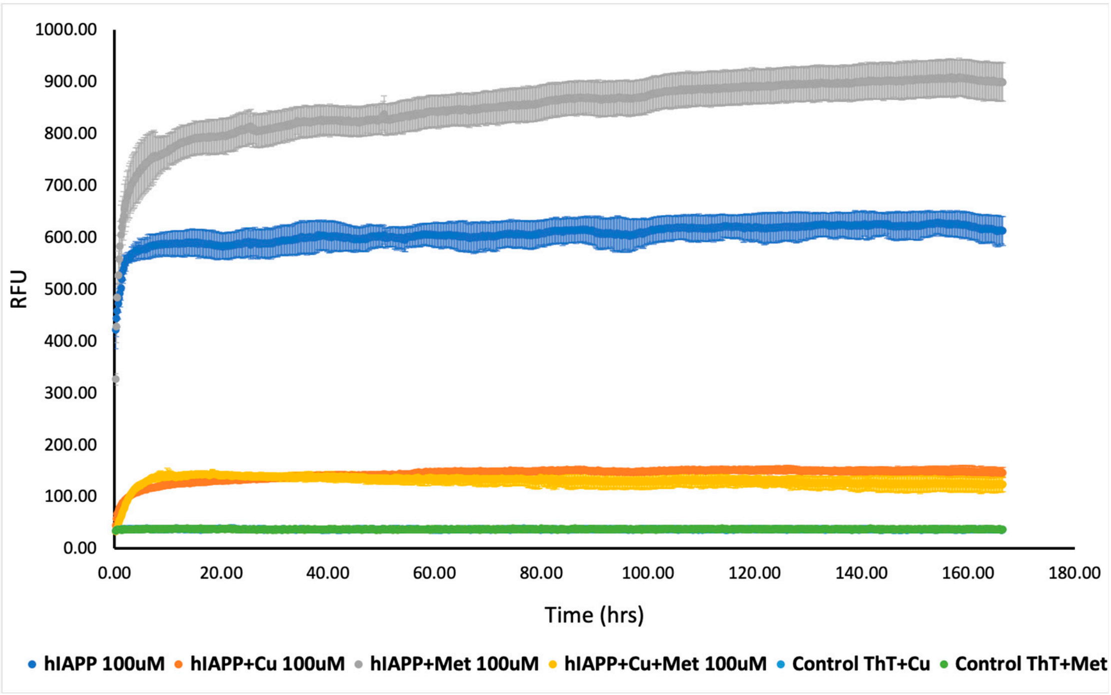

Figure S2. Effect of Cu (II) (100 μM) and Cu (II)/Metformin (100 μM; 1:1 molar ratio) on hIAPP (100 μM; in a 50 mM HEPES-buffered solution pH 7.4) fluorescence with ThT (20 μM) at 25 °C.

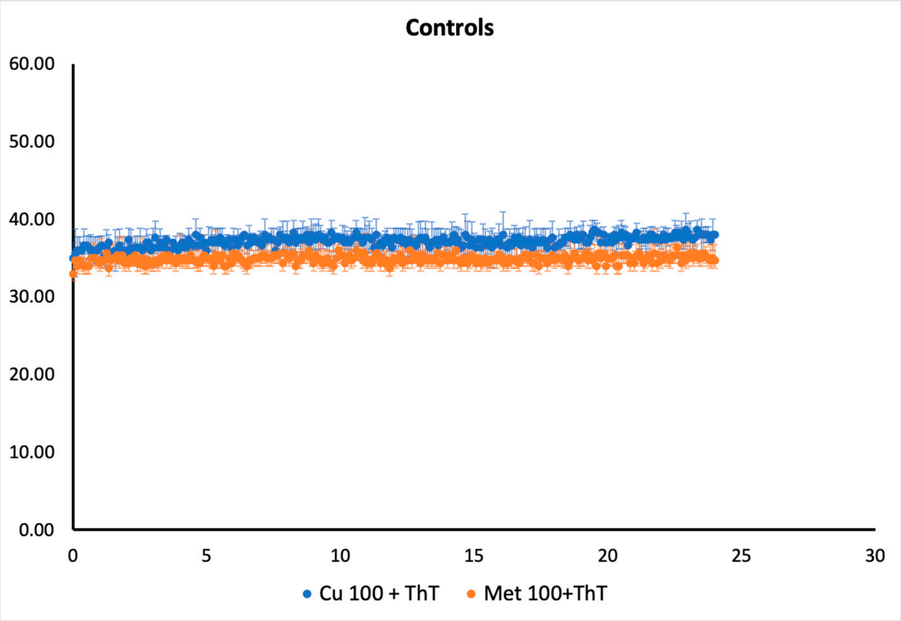

Figure S3. Effect of Cu (II) (100 μM) and Metformin (100 μM) on ThT (20 μM) fluorescence at 25 °C.

A

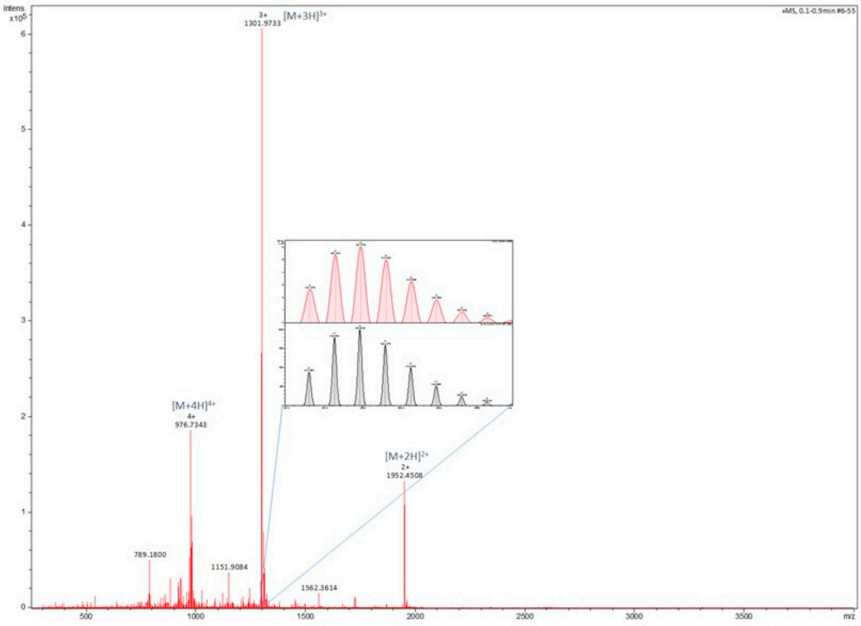

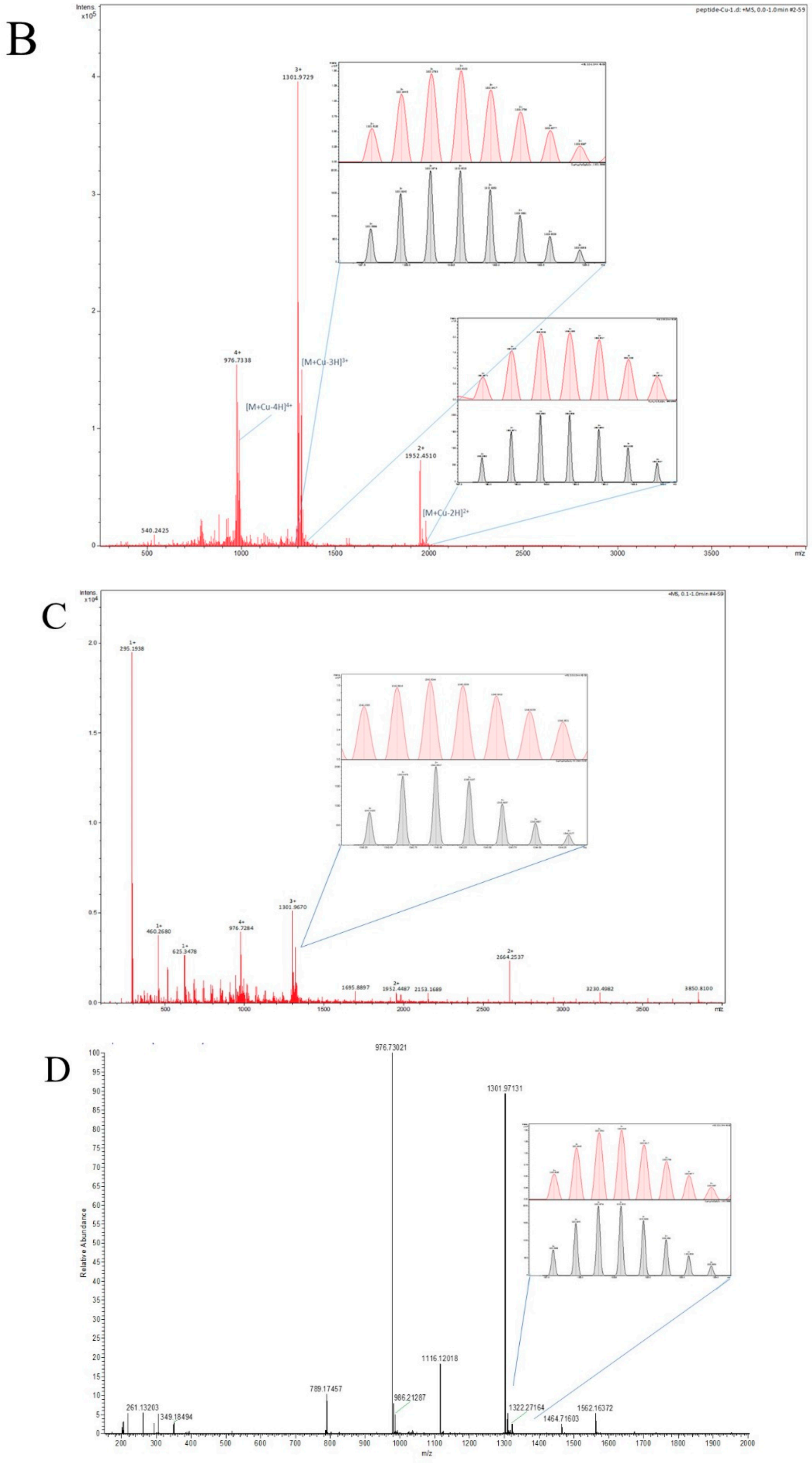

Figure S4. Full Scan Mass spectra acquired using the ESI-(+)-Micro-TOF-MS instrument. (**A**) hlAPPI solution. Signals at 976.7343; 1301.9733 and 1952.4508 *m/z* are assigned to hIAPP (M) at different protonation states: $[M+4H]^{4+}$; $[M+3H]^{3+}$; $[M+2H]^{2+}$; respectively. (**B**) hlAPPI/Cu(II) solution. Signals at 1322.6102 and 1983.4039 *m/z* are assigned to copper complexes: $[M+Cu(II)+H]^{3+}$ and $[M+Cu(II)]^{2+}$, respectively. (**C**) Metformin/hIAPP solution. Signal at 1342.9244 *m/z* is assigned to metformin (Met) adduct: $[M+Met-3H]^{3+}$. (**D**) Cu(II)/hIAPP/Metformin solution. Signal 1322.6102 is assigned to copper complexes: $[M+Cu(II)+H]^{3+}$. Insets: comparison of experimental (up) and simulated (down) isotopic distributions.

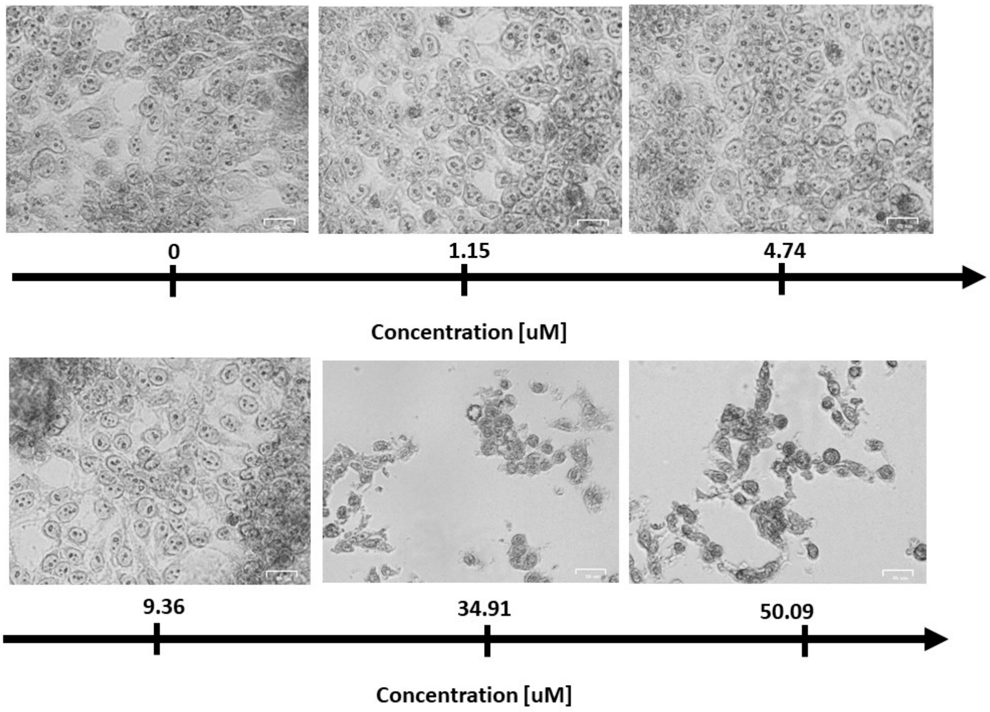

Figure S5. Live cell images of 293T cell cultures incubated 24h with growing concentration of hIAPP.

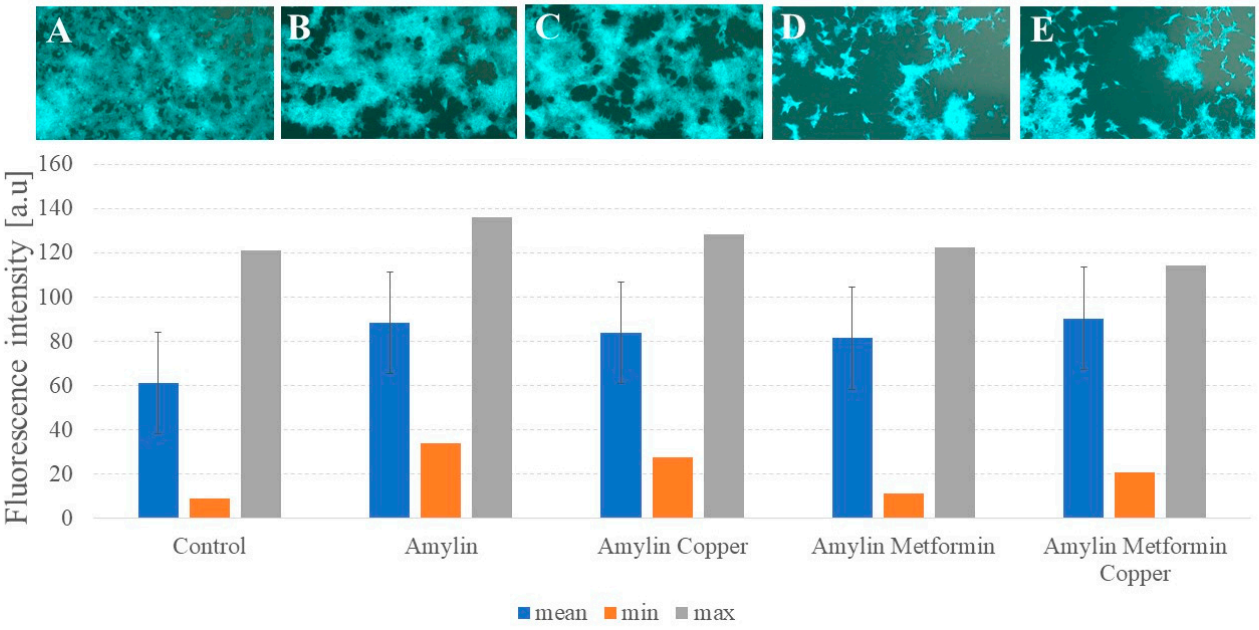

Figure S6. ThT live-cell fluorescence staining of 293T cells growing (**A**) in normal conditions; (**B**) with hIAPP (4.74 μM); (**C**) with hIAPP (4.74 μM) and copper ions (4.74 μM); (**D**) with hIAPP (4.74 μM) and metformin (4.74 μM); (**E**) with hIAPP (4.74 μM), copper ions (4.74 μM) and metformin (4.74 μM) for 24 h. The mean, min and max fluorescence intensity of each experimental sample (down) was calculated with ImageJ software.

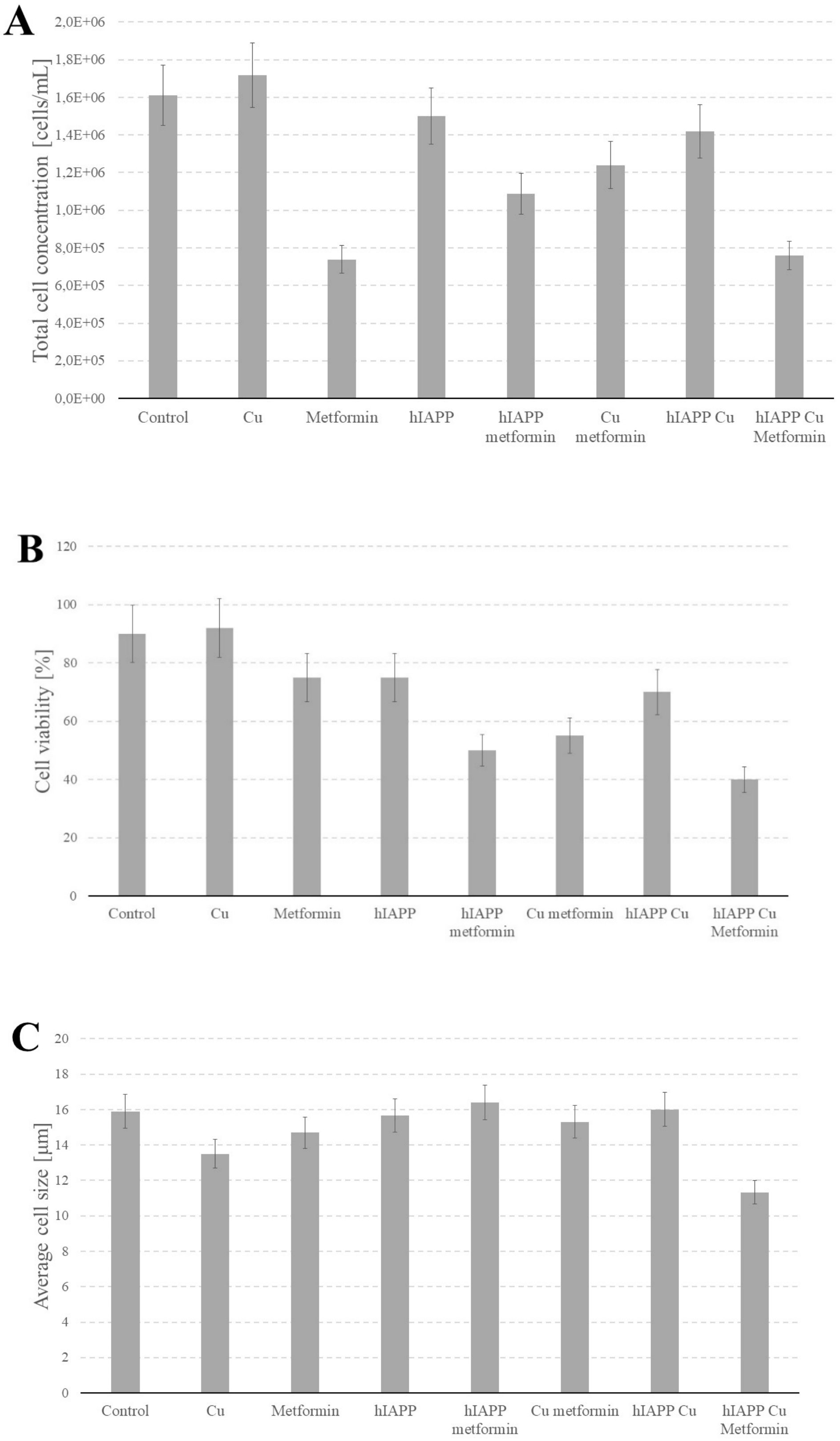

Figure S7. The graphical representation of LunaFL Cell Counter results of cell concentration (**A**), cell viability (**B**) and cell size (**C**).
